# Dr. Reynell Coates’ Contributions to Surgery, Note on the Means of Avoiding Excoriations of the Heel and Perineum in Fractures of the Lower Extremities, Treated by Permanent Extension

**Published:** 1842-05-14

**Authors:** Reynell Coates


MEDICAL EXAMINER.
NEW SERIES.
No. 20.] PHILADELPHIA, MAY 14, 1842. [Vol. I.

### *Contributions to Surgery.*

BY REYNELL COATES, M. D.

### *Note on the means of avoiding Excoriations of the Heel and Pirineum in Fractures of the Lower Extremities, treated by Permanent Extension.*

Continued from p. 277.

In order to avoid the difficulty of measuring the fractured limb, which constitutes one of the objections to the gaiter, the sock, and the ordinary handkerchief, and also to secure an absolute freedom from retraction of the inferior fragment under the tonic or spasmodic action of the muscles, I contrived, in 1820, a peculiar extending band, which was employed for several years in the Pennsylvania Hospital, though it appears to be no longer a favourite there. It consisted of a simple slip of stout Hollands, cut very slightly bias, about the breadth of three fingers in the middle, and two at either end, long enough to extend from the tendo Achillis around the malleoli, to form a cross on the extreme upper part of the instep, and descend on each side of the foot to a short distance below the sole. To the extremities of this strap were appended two pieces of strong broad tape, designed to be attached to the splint or cross board. A strip of soft, smooth buckskin of similar shape, and nearly of the same length was employed as a lining for the band, being secured to it by marginal stitches none of which were permitted to pass through the entire thickness of the leather. This last precaution is all important; for the direct action of a single stitch, like a crease or inequality in a muslin roller will, almost inevitably, occasion excoriation over the heel or instep, when pressed upon the skin for several weeks by even the most moderate extending or retentive force.

This band is employed in the same manner with the handkerchief, when used according to the first or common method, (p. 274.) It is equally inextensible; because the tapes act only upon the unyielding Hollands, and the buckskin is not stretched in the least. It does not embarrass admeasurement in the slightest degree; but it shares with the handkerchief the inconvenience of not acting exactly in the line of the axis of the limb, and when crossed too low upon the instep, it drags the toes forcibly downwards in like manner. The value of these objections has been fully discussed in the preceding section of this note, (pp. 275, 277.)

The motive for cutting the material of this band bias, is to avoid the sharp action of the edges upon the soft parts over the tendo Achillis; it permits the band to adapt itself more perfectly to the curvature of the surface, on which the principal pressure is exerted. But it is necessary that the obliquity of the threads should be very slight; so that many of them should extend through the entire length of the band; for, if cut very obliquely, the unyielding character of the material is lost, it stretches easily, is thrown into folds, and becomes altogether unmanageable. *When rightly made, and*

*carefully employed*, I think it superior in certainty of action to any other means of extension; but it should not be disguised that slight errors of construction or management will render it more likely than the handkerchief to produce ulceration: the latter is, therefore, safer in the hands of those who are not very frequently called upon to treat fractures of the thigh.

Hitherto, our attention has been exclusively confined to the causes of excoriation of the heel; but it is now time to speak of those affecting the perineum. This part of the body is scarcely less susceptible of injury from pressure or local excitement than the regions of the heel and instep; and, therefore, the presence of a stitch in contact with the skin, a rugosity in a bandage, or the long continued contact of adhesive plaster or oily dressings are nearly as active causes of irritation and ulceration in the former, as in the latter situation. To this we may add the fact, that inflammations of the perineum are peculiarly prone to extensive diffusion,—and even to the termination in gangrene, under violent stimulations or pressure,—in consequence of the looseness and compressibility of the sub-cutaneous cellular membrane.

But the perineum furnishes the only available point upon which the counter-extending force can be made to act in fractures of the lower extremities.* Hence, it is of the utmost importance that we should avoid all those sources of excoriation, in effecting counter-extension, which have been already pointed out when speaking of simple extension. There are but two essentially distinct means of applying the former power: 1st. The counter-extending perineal band—some form of which is common to all the plans and modifications of long splints founded upon the similar principles of Boyer and Dessault, with the exception of the apparatus of Dr. Hartshorne: and, 2d. The perineal cushion on the superior extremity of the internal splint adopted by the last named surgeon. This latter contrivance will come under our notice in a future note; but, as the remarks upon the general causes of excoriation and ulceration, which form the legitimate subject of the present paper, are equally applicable to both the above methods, it is convenient to confine our attention at present to the former only.

The counter extending bands in ordinary use are less various in structure than those designed for extension. Simple strips of muslin of considerable width folded or rolled upon themselves, long narrow sacks of the same material stuffed with bran or chaff, and a combination of handkerchiefs have been employed. The first and last are now considered as mere extemporaneous resources where a proper band cannot be immediately obtained; as in the country, at a distance from the office of the surgeon; or at sea in an ill-provided vessel. Both are liable to serious objection from their liability to catch the skin between the folds or creases of the material. This difficulty may be

* The short splints invented by Dr. Hutchinson, of this city, about half a century ago, which were designed to effect counter-extension below the knee by means of bands encircling the limb, have been long ago condemned by all experienced surgeons in this department of the art, in consequence of the embarrassment to the circulation produced by the apparatus; and it is my intention to discuss, in future notes, the merits of the method of counter-extension on the ham, as practised by the advocates of the double inclined plane, with its modifications from White to Amesbury; that by the weight of the body, as in the single inclined plane of Professor Gibson; that adopted by Hagedorn, and that of lateral pressure upon the pelvis, which constitutes the only effective power of Dr. Gibson's modification of the apparatus of Hagedorn.

removed by the careful application of a long strip of smooth buckskin between the band and the perineum ; but frequent attention is required to prevent the displacement of the leather, and its readjustment generally compels us to intermit the extension ; which is a serious disadvantage on many accounts. Yet I have seen several very successful cases that had been treated entirely by these contrivances without the production of much excoriation.

In the practice of this city, the elongated sack now takes precedence of the other counter-extending bands, but it is too generally made of extensible materials, and stuffed so lightly as to be very compressible ; both which defects render it impossible to prevent entirely the retraction of the inferior fragment of the broken bone during the first days of treatment. When made of common muslin, the pressure of the threads upon the perineum is very apt to produce excoriation, and, as there is necessarily a longitudinal seam upon one side of the band, if this should be turned towards the skin, ulceration will generally occur, unless a piece of buckskin or soft linen should be interposed.*

The following is a description of the counter-extending bands introduced by me many years ago, and well known to the profession in this city. They have been repeatedly described in former papers, and are of a nature to reduce the danger of excoriation of the perineum to a minimum.

Take a piece of brown Holland linen (not muslin) three and half inches in width, (for an adult) and long enough to extend from about six or seven inches above Poupart's ligament in front, around the perineum, below the tuberosity of the ischium, and thence upwards over the nates to the level of the summit of the sacrum. Double this strip in the direction of its width, and secure the edges by a firm longitudinal seam leaving about $\frac{1}{4}$ of an inch of selvage. Then revert the linen tube thus formed, so as to throw the selvage inwards, and secure one extremity of the tube to $\frac{3}{4}$ of a yard of tape, without puckering or irregular folds. Choosing this for the anterior part of your band ; determine how much of its length will be probably required to rest upon the front of the abdomen above Poupart's ligament when the apparatus is applied ; fill this with bran, not tightly packed, and secure it in place by basting across the tube, until you can quilt it down firmly and flat with saddler's silk, making one of the flattened sides to correspond with the longitudinal seam. In the next place, mark the probable length of that part of the band which will extend round the perineum, from Poupart's ligament fully to the tuberosity of the ischium ; pick out the basting, and proceed gradually to stuff this portion of the tube with bran driven down by a round stick about an inch thick, as firmly as possible, without endangering the bursting of the band or rendering it too inflexible for convenient application. Having accomplished this, fill the balance of the tube with unpacked bran ; attach a similar piece of tape to the posterior extremity ; close it, and quilt it like the anterior extremity. This band presents a solid, but flexible cylinder, of one inch diameter to the perineum, with flattened extremities, bearing the weight of the pelvis or pressing upon the abdomen; it is almost perfectly inextensible throughout, and, by the flatness of the ends, the skin is effectually secured from contact with the longitudinal seam. The material is also one of the least irritating that can be employed; but, by the ac-

*The sheepskin of the shops should never be employed in dressings applied immediately to the skin, as the alum which it contains acts as an irritant, soon producing an erythema and then a troublesome eruption.

tion of the perspiration and other accidents, the linen may become foul and the bran matted and hard. To remedy this evil, the round part of the cylinder should be inclosed in another tube, formed by lightly stitching together the edges of a strip of buckskin, face to face, without selvage; which is very easily done: and even this seam should be carefully turned from the perineum and scrotum when the tube is drawn over the cylinder. When occasion requires it, this buckskin tube may be replaced by another, without moving either the body or the limb of the patient.

The cotton and tow so frequently employed in stuffing counter-extending bands, are extremely objectionable, because they invariably become matted, irregular and knotty.

I am fully convinced that under proper attention to the hints given above, excoriation or ulceration of the perineum will never occur from the direct action of a counter-extending band, when the forces employed do not greatly exceed the necessary and warrantable amount.

*(To be continued.)*

